# Advances of mRNA vaccine in tumor: a maze of opportunities and challenges

**DOI:** 10.1186/s40364-023-00449-w

**Published:** 2023-01-18

**Authors:** Yuan Yuan, Fan Gao, Ying Chang, Qiu Zhao, Xingxing He

**Affiliations:** 1 Department of Gastroenterology, Zhongnan Hospital of Wuhan University, Wuhan, China; 2 Department of Gastroenterology, Tongji Hospital, Tongji Medical College, Huazhong University of Science and Technology, Wuhan, China; 3 Hubei Clinical Center and Key Laboratory of Intestinal and Colorectal Diseases, Wuhan, China

**Keywords:** mRNA vaccine, Tumor antigens, Algorithmic prediction, Sequence optimization, Adjuvants, mRNA delivery, Tumor immunotherapy

## Abstract

High-frequency mutations in tumor genomes could be exploited as an asset for developing tumor vaccines. In recent years, with the tremendous breakthrough in genomics, intelligence algorithm, and in-depth insight of tumor immunology, it has become possible to rapidly target genomic alterations in tumor cell and rationally select vaccine targets. Among a variety of candidate vaccine platforms, the early application of mRNA was limited by instability low efficiency and excessive immunogenicity until the successful development of mRNA vaccines against SARS-COV-2 broken of technical bottleneck in vaccine preparation, allowing tumor mRNA vaccines to be prepared rapidly in an economical way with good performance of stability and efficiency. In this review, we systematically summarized the classification and characteristics of tumor antigens, the general process and methods for screening neoantigens, the strategies of vaccine preparations and advances in clinical trials, as well as presented the main challenges in the current mRNA tumor vaccine development.

**Supplementary Information:**

The online version contains supplementary material available at 10.1186/s40364-023-00449-w.

## Introduction

In recent years, immunotherapy has gained significant momentum in the field of cancer treatment. One of favorable approach of cancer immunotherapy is tumor vaccines designed to stimulate the patient's adaptive immune system against specific tumor antigens, amplify and maintain specific T-cell responses, and tilt the combat between tumor and immune system in the immune direction to achieve control of tumor growth and eventual clearance [1]. However, traditional vaccine approaches such as live attenuated vaccines and inactivated viral vaccines that have achieved good preventive effects in a variety of infectious diseases are not suitable for tumor vaccine development [2]. Therefore, the development of more effective and versatile vaccine platforms has become an urgent need.

Over the past decades, significant technological innovations and research investments have brought several different vaccine platforms to the stage. Tumor antigens can be made into tumor vaccines through a variety of candidate vaccine platforms, including DNA, RNA, peptides, dendritic cells (DCs), viral vectors [3]. Although various vaccine platforms have their own characteristics, the choice of vaccine platform mainly depends on the security and efficacy of the vaccine, the speed and time as well as capital cost of preparation [4]. The successful development and widespread application of mRNA vaccines against SARS-COV-2 has led to the rapid maturation and optimization of the mRNA vaccine industry chain, and the experience gained has made the use of mRNA vaccine technology for tumor therapy promising. The advantages of vaccine platforms based on mRNA technology are: first, short development cycle: once the required mRNA sequence information is determined, rapid and large-scale production can be achieved by in vitro transcription with superior efficiency to traditional live or inactivated viral vaccines [3, 5]; second, dual immunity mechanism: in addition to the mRNA encoding antigens are capable of stimulating the immune response, the mRNA itself also has intrinsic immune stimulatory properties, and reasonable regulation of its immunogenicity allows it to function as an adjuvant [6]; third, efficacy: the modification of the sequence and the improvement of the delivery tools make the mRNA more stable and highly translatable [7]; forth, safety: unlike DNA vaccines and viral vectors vaccines, they do not enter the cell nucleus leading to the potential risk of insertional mutations in the host genome. Moreover, the in vitro preparation process can avoid the immunogenicity and cytotoxicity caused by virus-derived contaminants [8]. In addition, it can be degraded intracellularly by natural pathways [9].

In this review, we systematically described the key steps and technical reserves in the development of tumor mRNA vaccines, including the classification and characteristics of tumor antigens, general process and methods of neoantigen prediction and recognition, the latest strategies to improve the stability, translation efficiency and immunogenicity of mRNA vaccines, six common delivery tools and their respective functional characteristics, advances of relevant clinical trials completed or underway, as well as presents challenges that exist in current tumor vaccine development.

## The classification and characteristics of tumor antigens

**Fig. 1:**
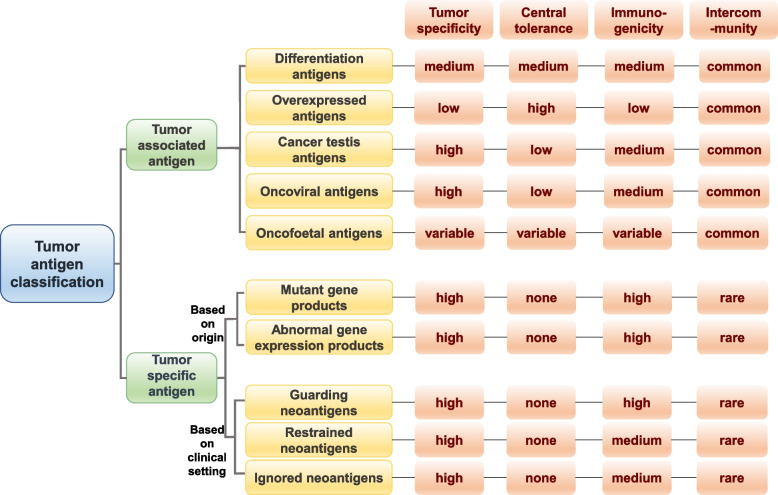
Tumor antigen classification and characteristics. Generally, tumor antigens are classified into tumor-associated and tumor-specific antigens. According to the expression levels and tissue expression characteristics, tumor-associated antigens can be further divided into overexpressed antigens, cancer testis antigens, differentiation antigens, oncoviral antigens and oncofoetal antigens. There are two ways to classify tumor-specific antigens, according to their origin, they can be classified as antigens derived from genetic mutations and antigens derived from abnormal expression regulation; according to clinical setting, they can be classified as guarding antigens, restrained antigens, and ignored antigens

Traditionally, non-mutated proteins that are highly expressed in tumors and low or no expression in normal tissues are defined as tumor-associated antigens (TAAs) [10], which could be further classified into the following types according to their expression levels or tissue expression characteristics (Fig. 1). The first category is overexpressed antigens which are expressed in normal tissues while up-regulated in tumor such as EGFR and HER2 [11]. These antigens are common, but have low tumor specificity and are subject to central tolerance, thus limiting their immunogenicity. Cancer testis antigens (CTAs) are another category of TAAs, which usually over expressed in a variety of cancers, as well as normally expressed in the normal testis and very few other tissues [12]. As the places where these antigens are expressed in the normal tissues is immune privilege site and doesn’t express major histocompatibility complex (MHC) I / II molecules, so it is considered to have high tumor specificity [12]. Another TAA widely presented is differentiation antigens, and these antigens are characterized by their expression in the normal and tumor tissues of the same origin [13]. Although these antigens such as PSA and gp100 are widely applied in the early development of tumor vaccines, the induced antigen specific immune responses may be compromised due to the central tolerance [14]. Oncofoetal antigens, such as 5T4, are upregulated during fetal development and in cancer, but rarely expressed in adult tissues [15]. However, another common oncofoetal antigen, CEA, is expressed in a variety of cancers, but also in non-tumor disease and normal colon tissue, and its expression level is also associated with aging [16, 17]. Therefore, it is difficult to generalize the tumor specificity, immunogenicity, and central tolerance of this class of antigens. The last type of TAAs are onco-viral antigens with the feature of "non-self", and thus have high tumor specificity and are not limited by central tolerance [18]. Several onco-viral antigens such as E6 and E7 oncogenic proteins have been used as vaccine targets for cancer prevention and treatment [19]. Nevertheless, a common problem of TAAs abovementioned as tumor vaccine targets is that there are also expressed to some extent in non-malignant tissues and may therefore fail to elicit an anti-tumor immune response due to self-tolerance mechanisms or produce off-target effects resulting in autoimmune toxicities [1, 19], so they are not the best choice for vaccine targets.

On the contrary, another type of tumor antigens is tumor specific antigens (TSAs) or neoantigens which are derived from somatic mutations, abnormal gene expression products characterized by “foreignness”, holding the great hope to elicit tumor specific T cell response [9]. Tumors exhibit a high rate of mutational accumulation various from single-nucleotide variants (SNVs), insertions or deletions (indels), gene fusion to splice variant, therefore creating great opportunities to generate TSAs [1]. Abnormal gene expression products generated by cancer-associated epigenetic changes, transcription, translation and post-translation abnormalities are also one of the important sources of neoantigens, but there are few relevant studies [9]. The first classification of tumor specific antigens is based on their origin. Recently, Franziska et al. proposed the theory of differentiation of neoantigens according to the clinical setting, linking them to dissection of the peculiar disease and therapeutic contexts and classifying them into three types [20]. The first type is named guarding neoantigen, which is characterized to stimulate of early antitumor immune responses in the absence of immunotherapy. Such neoantigens may have a guarding function reflecting in promoting early tumor rejection and regression before the formation of the tumor immunosuppressive microenvironment, slowing tumor growth, inhibiting metastasis, and preventing recurrence after operation of the primary tumor [20]. The second type of neoantigen is restrained neoantigens recognized by the reactivated T cells after ICB treatment with the feature of less antigenicity than guardian neoantigen, and it’s defined in terms of its predictive role of clinical benefits of immunotherapies such as ICB [20]. The third type of neoantigen is named ignored neoantigens. These neoantigens account for the majority of neoantigens generated by somatic mutations, but induced immune responses are undetectable before treatment until receiving vaccination. A crucial characteristic of these neoantigens is that the level of neoepitope presentation is insufficient to prime of naive T cells, but can be recognized by memory T cell [20]. This newly developed method of neoantigen classification is based on the characteristics of neoantigens before and after treatment, and endows the concept of "clinical" to previous neoantigens. Subsequently, this classification needs more experimental to support its classification value.

## Prediction of neoantigen candidates

The ideal tumor vaccine target is TSAs. Although there are huge advantages of neoantigen-based vaccines over the traditionally used TAAs, neoepitopes that can be recognized by spontaneously occurring T cells account for only 1% to 2% of somatic mutations of cancers, and their ability to mediate T cell-mediated tumor cell killing and anti-tumor effects varies, so there are still remaining difficulties to precisely make use of these neoepitopes [20, 21]. Identification of somatic mutation sites and prediction of binding affinity between neoepitope and MHC alleles are the two key prerequisites for optimally selecting neoepitope candidates [20, 22]. Although nonsynonymous mutations within cancer cells to be identified by whole-exome sequencing, significant obstacles in pinpointing which mutation-derived peptides can be correctly processed by antigen presenting cells and presented on the HLA molecules, as well as which one has strong immunogenicity and is not easily lost during immune editing remain to their success. Screening for suitable targets for vaccines has many aspects to consider, including properties of the neoantigen itself such as clonality and indispensability of mutant genes [23], dissimilarity to the wild-type sequence [24], transcript expression of neoantigens [25], MHC binding affinity [26] and the stability of the neoepitope–MHC complex [27] et al. The general methods and influence factors of neoantigen identification will be discussed in the following contents.

## Algorithmic prediction

**Fig. 2:**
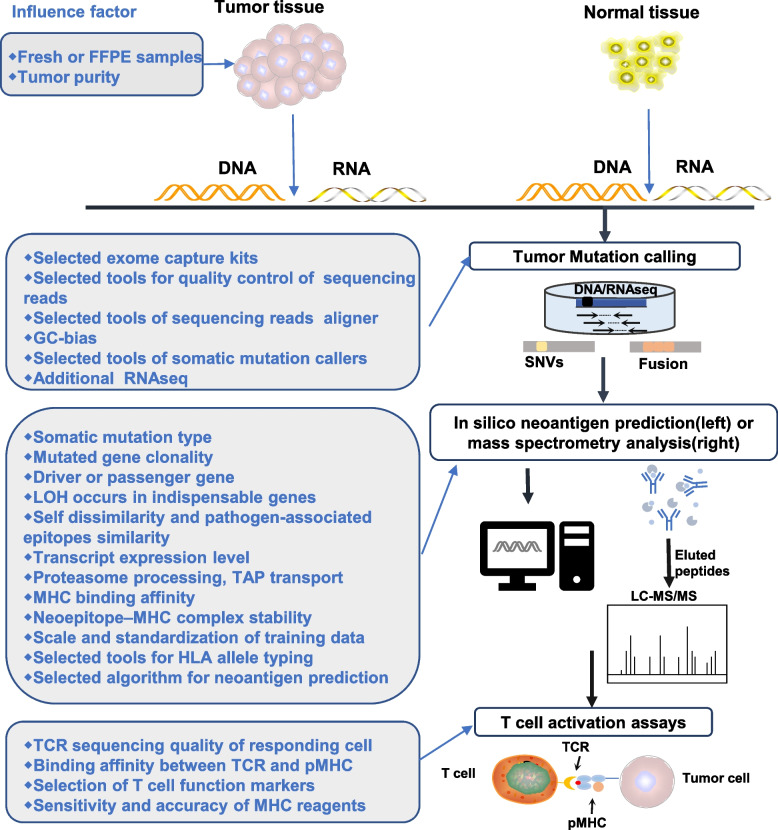
The general process of neoantigen identification and the influence factors of each step. Firstly, tumor tissue and normal tissue (usually para-cancer tissue or peripheral blood mononuclear cells) were sampled, their DNA was extracted for whole exon sequencing, and tumor samples were conducted RNA sequencing. Then, mutations are identified by comparing tumor sequences with normal sequences. Combined with predicted HLA typing, there are two methods to obtain neoantigens: neoantigen prediction pipelines (1) are used to predict neoantigens or selection of peptides that specifically bind to MHC molecules by mass spectrometry analysis (2). Finally, T cell activation assays are conducted to verify whether the selected neoantigens can specifically activate T cells. FFPE: formalin fixed paraffin embedded; SNVs: single-nucleotide variants; LC–MS: liquid chromatography and mass spectrometry; TCR: T cell receptor; pMHC: peptide major histocompatibility complexs; LOH: loss of heterozygosity; TAP: transporter associated with antigen processing

At present, the algorithm based on machine learning is still the mainstream method to predict neoantigens. A variety of parameters affecting the efficiency of neoepitopes presentation on MHC molecule are calculated by the current computational neoantigen prediction pipelines. The prediction process of neoantigens has been widely reviewed [28, 29]. Briefly, the general process of neoantigen identification including sample preparation, tumor mutation calling, neoantigen prediction by algorithm or identification by mass spectrometry (MS) analysis and validation of T cell activation (Fig. 2). During the prediction process, many factors influence the identification of mutated peptides presented by MHC molecule, and the common intrinsic and extrinsic factors that influence the ability of predicted neoantigens to activate T cells during neoantigen identification process are also shown in Fig. 2. Firstly, during the sample preparation, it is preferable to select frozen-fixed fresh samples rather than formalin fixed paraffin embedded (FFPE) tissue specimens to reduce the DNA damage [30]. In addition, the purity of the tumor also impacts the calling of gene mutations, which is generally required to be at least 80% for sequencing [31, 32]. In the sequencing stage, the selected exome capture kits, sequencing depth, quality control, alignment with reference genome, and correction of sequencing read coverage in GC-rich regions will all impact the final result of mutation identification [33, 34]. Besides, the level of transcript expression and degradation rate of mutant peptides are closely related to whether they can be candidate epitopes. Mutated peptides with high expression at the protein level are more likely to be captured by MHC molecule in a quantitative manner even if with low binding affinity [25, 35]. This is why many studies took transcript expression level as one of criterion for screening mutated gene [36]. Although tumor mutation resources are abundant, in the escape stage of immune editing process, tumor cells will escape the surveillance of the immune system through mechanisms such as loss of tumor antigen expression to proliferate and invade [37]. Therefore, the type of mutation from which the neoantigen originates, whether it is a clonal mutation, whether it is a driver or passenger gene, and whether exsiting loss of heterozygosity will all influence the durability of neoantigen expressed on tumor cells [23, 38, 39]. The presentation of epitopes is restricted by HLA molecules. Therefore, HLA allele typing is required to predict potential immunogenic neoepitopes. Tools that commonly used in the HLA typing include PHLAT, Optitype, Polysolver and RNA2HLA et al. [40–43]. Most of the developed neural network tools are used to predict binding affinity of a large number of MHC class I alleles for given neoantigen candidate sequencing [32]. These algorithms are trained based on wet laboratory binding affinity data or eluting mutant peptide ligand data detected by mass spectrometry, and are continuously optimized [20, 26, 32]. A typical example is that NetMHCpan and MHCflurry achieved the best performance in the evaluation system with the area under the ROC curve (roc- AUC) as the performance index [26, 44]. It is considered that the mutant peptides are more likely to induce CD8 + T cell response when the predicted HLA binding affinity is above medium (IC50 < 150 nmol/l) [45]. Some tools like NetChop and NetCTL as well as NetCTLpan also incorporate predictors of proteasome processing, transporter associated with antigen processing (TAP), and MHC binding to give a comprehensive score of each peptide's inherent potential as a T cell recognized epitope [46, 47]. Calis JJ et al. designed a model cable of utilizing amino acid properties and their positions in peptides to predict the immunogenicity of class I peptide MHC (pMHC) complexes and the relative ability of pMHC complexes to initiate an immune response for further facilitating the screening of optimal neoantigens [48]. In addition, the stability of the interaction between mutant peptide and MHC molecules appears to be more important than the MHC binding affinity in the immunogenicity prediction process. For this reason, Dylan T Blaha and his colleagues used a rapid, high-throughput method to experimentally determine peptide/HLA thermal stability, completing the guidelines for neoantigen selection [27].

So far, the epitope prediction methods mainly focus on the MHC I binding epitope, as the MHC I peptide binding groove has a closed end, and most of its binding epitopes are sequences containing 8–11 amino acids. In contrast, the end of groove for MHC II binding peptide is open, and the binding property is not strict, which means that it has ability of binding and presenting longer and more variable length peptides [1]. It was found that in addition to CD8 + T cells, specific CD4 + T cells play an indelible and even dominant role in inhibiting tumor growth, which is related to the open MHC II molecules binding peptide groove having the opportunity to recognize more peptides [49–51]. However, there are few algorithms for MHC II molecular binding peptides as peptide binding by HLA-II is more promiscuous than HLA-I which makes it more challenging to identify binding motifs. There are tools such as EDGE and MARIA that combine the prediction of HLA I and HLA II binding peptides [52, 53]. EDGE predicted tumor specific neoantigens have also been developed into personalized tumor antigen vaccines and some clinical trials have been carried out in in melanoma, gastrointestinal cancer and breast cancer [20, 22]. MARIA is a multi-mode recursive neural network based on deep learning for predicting neoantigen epitopes restricted to specific HLA Class II alleles [53]. Common tools with their functions or characteristics required for each part of the antigen prediction process including quality control of sequencing reading, read alignment, somatic mutation calling, HLA allele typing and neoantigen prediction are shown in Table 1.

**Table 1:** Common tools with their functions or characteristics in the neoantigen prediction process

Neoantigen prediction step	Common tools	The function or characteristics of tools
**Quality control of sequencing reading**	FastQC [162]	To analyze samples with uncertain DNA sources or multiple sources
	ClinQC [163]	Quality control and modification of raw sequencing data generated by Sanger sequencing, Illumina, 454 and Ion Torrent sequencing
	Lighter [164]	A commonly used and efficient tool for correcting sequencing errors
	Musket [165]	An efficient correction tool for Illumina short-read data
	SequencErr [166]	An emerging tool for evaluating, calibrating, and monitoring sequencer error rates
**Read alignment**	NovoAlign [167]	NGS aligner; high sensitivity towards short reads, long reads and complex genome; slow alignment; high percentage of properly paired reads
	BWA [167]	NGS aligner; low sensitivity towards short reads; fast alignment; high percentage of properly paired reads
	Smalt [167]	NGS aligner; low sensitivity towards short reads; medium alignment speed; low percentage of proper pair in both short and long reads
	Stampy [167]	NGS aligner; moderate sensitivity towards short reads; slow alignment; high percentage of proper pair in both short and long reads
	Bowtie2 [167]	NGS aligner; low sensitivity towards short reads; medium alignment speed; low percentage of proper pair in both short and long reads
	STAR [168]	A universal RNA-sequence aligner with superior mapping speed
**Somatic mutation** **calling**	VarScan 2 [169]	Discover SNVs and CNVs
	VarDict [170]	Discover SNV, MNV, InDels, complex and structural variants
	SomaticSniper [171]	Discover somatic point mutations
	MuTect [172]	Discover somatic point mutations with very low allele fractions
	cn.MOPS [173]	Detection of CNVs
	Manta [174]	Discover structural variants and indels
	FusionMap [175]	Detect gene fusions from RNA-Sequence or gDNA-Sequence
**HLA allele typing**	PHLAT [40]	High accuracy at four-digit (92%-95%) and two-digit resolutions (96%-99%)
	OptiType [41]	High two-digit accuracy (97%), only serves for HLA class I typing
	HLA-HD [176]	Determine with 6-digit precision
	HLA-VBSeq [177]	Determine with 8-digit precision
**Neoantigen** **prediction**	NetMHCpan [178]	MHC-I binding prediction
	NetMHCIIpan [178]	MHC-II binding prediction
	MHCflurry [44]	MHC I binding prediction; faster prediction than NetMHCpan
	DeepHLA-pan [179]	Prediction of HLA-peptide binding (binding model) and the potential immunogenicity (immunogenicity model) of the peptide-HLA complex
	NetCTL [180]	Prediction of proteasomal cleavage, TAP transport efficiency, and MHC I affinity
	EDGE [52]	Prediction of HLA I and HLA II binding peptides
	MARIA [53]	Prediction of HLA I and HLA II binding peptides
	ATLAS [22]	Using patient’s T cell immune response machinery to identify optimal tumor-specific neoantigens

SNV: single nucleotide variation; CNV: copy number variation; MNV: multi-nucleotide variation

Although the algorithm prediction has developed rapidly and made great breakthroughs, in consideration of randomness in mutation and complicated situation in tumor microenvironment, and it’s hard to be denied that there is still a long way to go before it is widely used. At present, there is still no consensus on many aspects of neoepitope prediction. Another obstacle is that the training data sources used by different algorithms have problems of sample size, uniformity and standardization, which may lead to different epitopes predicted by different algorithms and lack of screening criteria.

Besides, another dilemma is that in the process of epitope prediction, there is a lack of a standard to calculate the weight of various influencing factors, so as to comprehensively prioritize the candidate neoepitopes, which inevitably makes users subjectively select candidate antigens for follow-up work, greatly increasing the inaccuracy of the algorithm and the workload of users.

## Experimental approach

In general, experimental discovery of neoepitopes relies on next generation sequencing with mass spectrometry based immunepeptiomics. Immunopeptiomics is the collective identification and quantification of sample-specific repertoires of HLA-presented peptides with the rationale of isolating MHC molecules from cells following with immunoaffinity purification and detection by liquid chromatography, matching MS data to a specific reference database that included somatic mutation information [54, 55]. Current mass spectrometry techniques make it possible to identify a large number of pMHC complexes, regardless of whether they are provided from cell lines or patient's tumor tissue [29]. In an attempt to identify neoepitopes in two common mouse tumor models, Mahesh Yadav et al. developed a method combining whole-exome and transcriptome sequencing analysis with mass spectrometry, coupled with an pMHC complex structure prediction algorithm, and ultimately discovered and validated three immunogenic neoepitopes that capable of triggering strong immune responses in MC-38 tumor-bearing mice [36]. HLA type of human species is more diverse and complex than mouses’. In a study to identify neoepitopes in human, Michal Bassani-Sternberg performed mass spectrometry analysis of 95,500 peptides isolated from patient with melanoma. in addition to 4 of 11 mutant ligands were shown to be immunogenic to trigger neoantigen-specific T cell responses, specific effector T cells targeting the MS-identified neoantigens were also detected in patients' tumors and peripheral blood [56]. Discovering the HLA bound ligandome by mass spectrometry is hopeful for developing tumor vaccines. Still, the precise identification of immunogenetic neoepitopes remains a variety of challenges. For instance, the current mass spectrometry techniques suffer from false negatives due to the inability in fully identifying neoepitopes which could be recognized by T cells [29]. However, such failure of recognition cannot only be attributed to the low binding affinity between neoepitopes and MHC molecule, because some epitopes with low predicted binding affinity can be detected by mass spectrometry [29]. Therefore, other factors affecting the activation of T cell response by epitopes should also be taken into consideration to improve the false-negative situation. Immunopeptidomics often requires a large quantity of tissue samples, which can affect its accuracy if not enough material is collected, as well as interfere with the accurate identification of the cancer immunopeptidome when the tumor sample has a large stromal content [57]. Therefore, Alice Newey and colleagues established an in vitro model of patient-derived organoids (PDOs) in colorectal cancer and demonstrated the feasibility of MS-based immunopeptidomics for CRC PDOs [57]. Wenwen Wang and colleagues also developed hepatobiliary tumor organoids and performed multi-omics analysis based on this model to validate the feasibility of screening for neoantigen-peptides [58]. The development of organoid models not only enriches preclinical models for identifying neoantigen-peptides, but also can be used to study how drug treatment or cytokines affect the expression of HLA molecules, and the number of neoantigen-peptides. Besides, as intra-tumoral HLA II molecule is mainly expressed by antigen presenting cells (APCs) rather than cancer cells, this may lead to inaccurate outcome of MS analysis for detecting peptides on APCs in the tumor microenvironment. Therefore, Jennifer G. Abelin et al. developed a technique for discovering HLA II binding motifs to improve sensitivity of MS analysis in discovering the HLA II epitopes [59]. The further method to detect whether the MHC molecule presenting neoantigen stimulates T cell response is based on T cell detection system. T cell-based detection system is inseparable from the detection of T cell functional phenotype, such as according to IFN- γ production or CD137 expression to determine whether it is activated or not [60]. T cells can also be detected by MHC reagents loaded with a series of labeled neoantigens such as in situ MHC tetramer (IST) [61, 62]. One application related to this type of detection method is ATLAS™, an emerging technology platform that recognizes tumor specific neoantigens through the patient's T cell immune response mechanism, with a considerable amount of clinical or preclinical researches underway [22].

However, both regular mass spectrometry and T cell response assays are at the limit of low throughput of capacity and false negatives of sensitivity. In addition to standardizing the detection methods and formulating reasonable and unified inclusion comparison standards, it is also necessary to compare the database of immunogenic neoepitopes identified by mass spectrometry and T cell assays, so as to better understand the key factors of the immunogenicity of neoepitopes and optimize the neoepitope screening strategy.

## Current status of mRNA tumor vaccine technology development

**Fig. 3:**
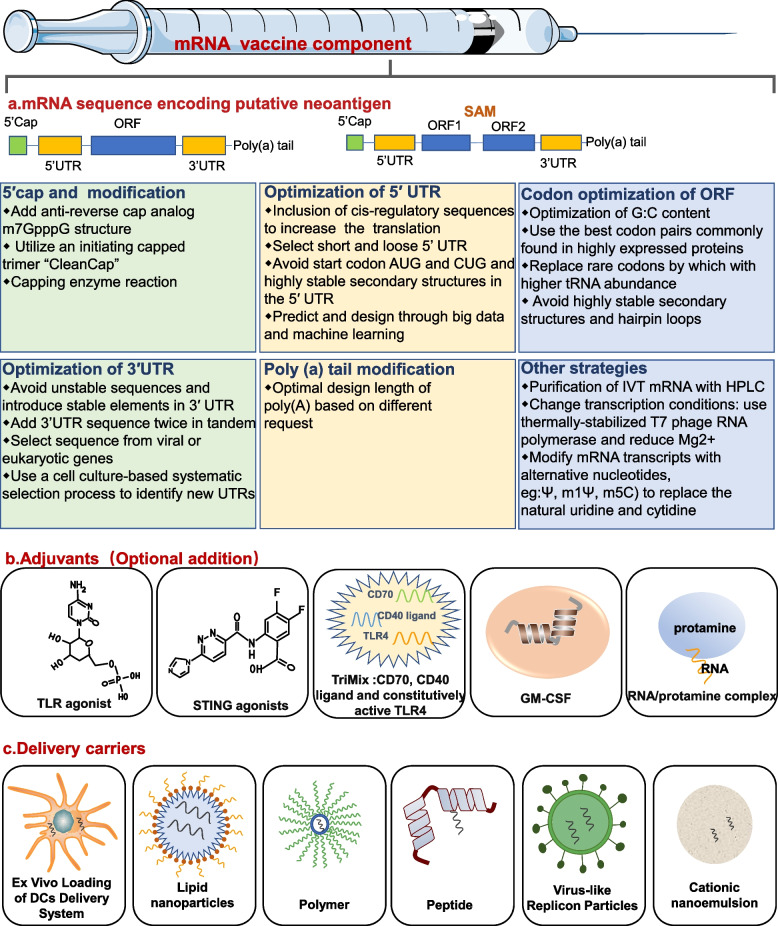
The main components of mRNA tumor vaccine. The first component is mRNA sequence encoding putative neoantigen, which could be either non-replicating or self-amplifying. The boxes below show methods to improve mRNA translation efficiency, stability, and immunogenicity. The second component is several common adjuvants that can be added selectively. The third component is several common mRNA delivery tools. UTR: untranslated region; ORF: open reading frame; SAM: self-amplified mRNA; TLR: Toll-like receptor; STING: stimulator of interferon genes GM-CSF: granulocyte–macrophage colony-stimulating factor; DCs: dendritic cells

mRNA, also known as messenger RNA, was first discovered by Brenner et al. in 1961, and it is a single stranded RNA transcribed from a strand of DNA as a template and carries genetic information to guide protein synthesis. In the 1990s, in the context of a series of breakthroughs in vitro transcription technology represented by Wolfe et al. whom injected an in vitro transcription (IVT) mRNA expression vector into mouse skeletal muscle and detected the protein expression for at least 2 months [63], a new milestone concept that regards mRNA as a potential therapeutic tool especially as vaccine platforms was born. The principle of mRNA vaccine is to transfect the corresponding transcripts which carrying tumor antigen information into the cytoplasm of host cells (usually APCs) after confirming one or more target proteins. The encoded proteins of interest can be displayed on the surface of APCs through binding with MHC molecules to activate the immune system, including B cell-mediated humoral response and CD4 + T / CD8 + T mediated cell response [19, 64]. For the batch synthesis of mRNA, the most efficient method is IVT. IVT primarily utilizes linear DNA as a template with aid-assisting by bacteriophage RNA polymerase to prepare mRNA. The main process steps include transcription of linearized plasmid DNA into mRNA, chemical modification (including 5'-end capping structure and 3'-polyA tailing structure), separation and purification process [5]. Early applications for mRNA vaccines were non-replicating, unmodified mRNAs. This kind of naked mRNA not only has strict storage conditions and high cost, but also is easily degraded rapidly by extracellular RNases and cannot be effectively internalized by APCs. Additionally, it also activates downstream interferon-related pathways to stimulate innate immunity resulting in accelerating its degradation [3, 65, 66]. This unstable characteristic greatly restricted the early development of mRNA vaccine. In order to overcome the obstacles, researchers developed modified mRNA and virus-derived self-amplified mRNAs (SAM) to enhance their stability and accommodate their immunogenicity (Fig. 3). The specific strategies are discussed below.

## Optimization of mRNA translation and stability

### 5′ cap and modification

The 5′cap is a protective structure, containing a m^7^G at the 5' end of eukaryotic mRNA sequence connected to the first nucleotide with a 5′ 5’-triphosphate bridge named m7GpppN, which is also referred as cap 0 structure [67]. The 5’cap structure can enhance the stability and translation efficiency of mRNA, which is reflected in its ability to eliminate the free phosphate groups on the sequence, regulate the pre-mRNA splicing, protect RNA from exonuclease cleavage, bind with eIF4E to initiate translation and recruit the required proteins [5]. Therefore, a well-formed 5′cap is critical to IVT mRNA to function. Common capping strategies for IVT mRNA include enzymatic and chemical methods. The most representative enzymatic method is the Vaccinia capping system, which is used in the second step following the initial synthesis of IVT mRNA, mainly utilizes Vaccinia virus Capping Enzyme (VCE) that combines enzyme activities of RTPase, GTase, and G-N7 MTase to synthesize the CAP 0 structure [5, 68]. The 5′cap structure produced by this method is most similar to that of natural eukaryotic mRNA, and the capping efficiency is very high as well as is unacted on the length, sequence and substrate structure of RNA [69]. Another common method is to add a cap analogue (m7GpppG) to the 5’ end of the mRNA through bacteriophage polymerases during transcription, minimizing the number of reaction steps and enzymes required to make mRNA [70]. But this chemical method risks the binding of the cap analogue to the mRNA in the opposite direction, which will result in a decrease in the translation efficiency [71]. Anti-reverse cap analog (ARCA) was developed to overcome reverse cap analogs by chemically modifying the m7G moiety with 3-OH or 2-OH to successfully achieve forward incorporation of cap analogues, thereby increasing the translation efficiency [72]. Additional methylation of 5′cap endows its structural and functional versatility. Compared to cap 0, cap 1 is characterized by a methylated 2′-OH on the first nucleotide and cap 2 is characterized by a methylated 2′-OH on the second nucleotide, which serves a useful role for body to discriminate "self" and "non-self" RNA, thus preventing the mRNA translation from inhibition [67]. Processing of cap 0 into a cap 1 structure can be achieved by using 2'-O-methyltransferase to catalyze the transfer of a methyl group from s-adenosylmethionine to IVT mRNA with cap 0 [73]. For now, the cap1 structure is most commonly used for capping mRNA vaccines. However, co-transcriptional reactions with ARCA require a high level (from 4:1 to 10:1) ratio of ARCA to GTP, yet capping efficiency remains low (60–80%) and at least 20% of mRNAs fail to be capped after transcription [67]. The CleanCap™ developed in 2018 utilized an initiating capped trimer to enable co-transcribed mRNA to acquire cap1 structure, overcoming the limitations of previous capping methods [70]. Besides, another trinucleotide cap analogue with a LNA moiety synthesized by Annamalai et al. further improved the translation efficiency of mRNA, but its effect on the stability of mRNA needs to be further investigated [74].

## Modification of 5ʹ- and 3ʹ-UTRs

The 5’-UTR and 3’-UTR are located on the flanks of the coding region, respectively, and contain various regulatory sequences that affect the stability, translation and localization of mRNA [73]. The cis-regulatory element in UTRs regulates the translation and half-life of mRNA. Currently, designed 5 ' UTR sequences are mainly derived from genes such as globin and Hsp70, or predicted and designed through big data and machine learning [75–77]. The rational design of 5 ' UTR should avoid the appearance of the start codon (AUG) and (CUG) in 5 ' UTR which may probably disturb the translation process [64]. Secondly, the secondary structure of 5'UTR also influences the initiation efficiency of the suboptimal start codon, thus it is also recommended selecting short and loose rather than a highly stable secondary structure during the design [64, 78]. On the other head, the 3' UTR sequence is mainly obtained from genes such as hemoglobin subunit α (HBA) and hemoglobin subunit β (HBB) [79]. When designing and synthesizing UTR sequences, attention should be taken to avoid introducing too many unstable sequences, and to regulate mRNA degradation by adjusting AU-enriched sequences and GU-enriched sequences [80–82]. In addition, stringing together two random 3’ UTRs containing stable elements has also been shown to improve the translation efficiency [83]. Moreover, Alexandra G and colleagues developed a novel cell culture-based approach to screen for cell type-specific 3’ UTR elements that contribute to mRNA stabilization and found that such 3’ UTR has more protein expression products than mRNA with the b-globin 3'UTR [83].

## Codons modification in the ORF

The influence of non-coding regions alone on the translation and stability of mRNAs cannot fully explain the large number of half-life changes observed in the entire transcriptome [84, 85]. It was found that codons in the ORF may have a general impact on the transcriptome translation and stability, thus the concept of codon optimality, which referred as the inhomogeneous decoding rate of codons by ribosomes, was introduced to represent a measure of translation efficiency, which has a broad and powerful effect on mRNA stability [85]. Replacing non-optimal codons with optimal codons significantly enhances mRNA stability, translation speed and the protein yield [85]. However, it is not advisable to select optimal codons solely, as some of the suboptimal or infrequent codons serve to slow down the translation of critical structural motifs to ensure proper protein folding [86]. Secondly, codon bias is closely related to the percentage of GC content within the OFR, so adjusting the GC content in the OFR will also alter the translation elongation rate [87, 88]. Besides, the introduction of stable RNA secondary structures near the start codon should be avoided since they require more energy to unfold before translation initiation, which slows down the translation rate [89, 90]. In conclusion, multiple factors should be considered comprehensively to achieve regulation of translation rate by optimizing codons of ORF, rather than simply believing that a higher translation elongation rate is better.

## Poly (A) tail modification

The poly(A) tail is a major regulator of gene expression, regulates the mRNA translation by synergistically acting with the (m7G) cap of 5’-end, and is also involved in regulating mRNA stability [91]. The poly(A) tail can be added to the 3 'end of the mRNA by either Recombinant Poly(A) polymerase-mediated polyadenylation or by transcription according to a designed DNA template, and the major difference between the two methods is that the former Poly(A) tail length is fixed, while the latter allows for the regulation of Poly(A) tail length as needed [5, 92]. It has been demonstrated that the mRNA translation is largely controlled by length of the poly(A) tail. Previous studies have suggested that the tail length is positively correlated with the mRNA translation and stability, and indeed some highly translated and stable RNA has been shown to have a short poly(A) tail (less than 30 nucleotides) [93, 94]. In addition, the M7G cap at the mRNA 5’ end can form a closed loop with the poly (A) tails through the interaction with/between eIF4E, eIF4E and PABPC molecules and communicate directly [95]. Such cap-tail combination can significantly improve the translation efficiency, but at the same time, there is a risk that translation termination occurs near the mRNA 5ʹ end [91].

## Modulation of immunogenicity

In addition to mRNA encoding antigens that activate the immune system, due to mRNA itself has exogenous resemblance to infectious pathogens, it can prompt pattern recognition receptors to sense pathogen-associated molecular patterns, activate DCs to initiate signals to produce pro-inflammatory factors via endosomal anchored receptors such as toll-like receptors in immune cells and cytokines via cytosolic sensors including cGAS, RIG-I and MDA5 in non-immune cells, and ultimately leading to innate and adaptive immune responses [3, 96, 97]. Undoubtedly, the inherent immunostimulatory properties of exogenous mRNAs play an adjuvant-like role in driving dendritic cell maturation, which is beneficial to a certain extent for vaccination. Nevertheless, excessive innate immune sensing of mRNA can produce a large amount of interferons such as type I IFNs leading to translation stagnation, degradation of RNA and serious systemic side effects such as autoimmunity, which can negatively affect the immune response [98]. How to maintain the balance of proper activation of innate immunity has been continuously explored. Currently, purification of IVT mRNA, changing transcription conditions, adjustment of mRNA sequence, addition of additional adjuvants and other strategies are widely used to shape the immunogenicity of IVT mRNA.

## Purification of IVT mRNA

One reason for the strong immunogenicity of IVT mRNA is that contaminants generated during the production process include double-stranded RNA (dsRNA), short transcripts are not effectively removed, and these byproducts can lead to high IFN-1 production by activating innate immune receptors like RIG-I and MDA5 [6]. For small-scale mRNA preparation, natural PAGE or agarose gel electrophoresis can identify the correct ssRNA [99]; for large-scale mRNA preparation, purification by reversed-phase high performance liquid chromatography (HPLC) can effectively separate IVT byproducts and improve protein production, but this method is costly and cannot prevent high levels of cytokine secretion [100]. Another economical alternative to HPLC is using cellulose to bind dsRNA and separating it from the ssRNA mobile channel in the spin column to remove dsRNA [101]. The dsRNA clearance rate and purified mRNA translation rate of this method was comparable to that of HPLC, but purification of nucleotide modified mRNA rather than unmodified mRNA was required to effectively prevent high levels of IFN secretion [6, 101].

## Transcription conditions

In addition to removing dsRNA in the IVT process, dsRNA byproducts can also be reduced from the source by optimizing the transcription conditions. Compared with synthesizing mRNA under moderate conditions (37 °C), the synthesis of mRNA at 55℃ using thermally-stabilized T7 phage RNA polymerase can effectively reduce dsRNA produced by 3' -extension of run-off products, while the poly(A) tail can reduce the dsRNA produced by the antisense RNA, thus the combination of both methods can effectively reduce the formation of both dsRNA byproducts from both sources [6]. Moreover, the level of dsRNA can be effectively reduced by reducing Mg^2^ + in transcriptional reaction conditions, and this is a ubiquitous phenomenon, independent of the template of DNA sequence, structure and length [102].

## Nucleotide modification

Another strategy for regulating immunogenicity is nucleotide modification, which has been described in some detail in other reviews [6, 7]. Briefly, natural nucleotides are modified into pseudouridine (ψ), N6-methyladenosine (m6A), 5-methylcytidine (m5C), 2-thiouridine (s2U) and N1-methyl pseudouridine (m1ψ), which can label RNA "self" and avoid innate immune system recognition of IVT mRNA [103, 104]. The basic mechanism can be summarized as follows: ψ, M1 and m5C avoid the conformation change of RIG-I after binding with mRNA [105]. ψ, m6A, m5C and s2U can make mRNAs not bind to TLR3, TLR7, TLR8 and RIG-I [106, 107]. By using ψ to replace U, RNA molecules can avoid the activation of OAS and downstream RNA enzyme L leading to RNA cleavage [108]. Many studies support that nucleotide modification can enhance mRNA stability and the translation efficiency, as well as reduce immunogenicity. For example, nucleotide modification with m5C and ψ can increase mRNA uptake and protein expression, as well as decreased the formation of stress granules, which served as a marker for innate immune activation [109]. But this is not absolute, as some studies also indicate that unmodified mRNA has better protein yield than ψ substituted mRNA, and its induced cytokine production is also very slight [110]. Another study also found no substantial difference in the protein expression and cytokine secretion from unmodified or ψ substituted mRNAs delivered intravenously through lipid nanoparticles (LNPs) [111]. Besides, different cells may show different responses to the same modified nucleoside, such as different pro-translation effects [6]. Therefore, the variety of nucleoside modification methods and effects, the benefits and potential hazards of nucleoside modification must be comprehensively considered before implementation.

## Additional adjuvants

The strategies described above focus on reducing the immunogenicity of IVT mRNA, but delayed activation of innate immune responses can adversely affect protein translation, antigen expression and processing as well as T cell activation, thereby compromising vaccine effectiveness. Therefore, an increasing number of studies have attempted to add additional adjuvants to vaccines. The first is TLR agonist, which has been developed to activate TLR3, TLR4, TLR5, TLR7 and TLR9, most of which is still in clinical trials, among which MPLA as a TLR4 agonist and imiquimod as a TLR7 agonist has been approved by FDA for infectious diseases or cancer [112, 113]. The second is STING agonists, which can currently be delivered by nanoparticles. A study using liposomal nanoparticles to deliver cGAMP in a mouse model of breast cancer found enhanced expression of MHC II and costimulatory molecules [114]. In another study, STINGVAX, a kind of tumor vaccine, was developed by co-formulating STING agonist CDNs as an adjuvant with vaccine cells expressing GC-SF. Its application in a mouse melanoma model was observed STING-dependent expression of IRF3 and type I IFN and tumor regression [114, 115]. The third adjuvant, TriMix, is a cocktail mRNA encoding three proteins, CD40 ligand (CD40L), CD70 and constitutively active TLR4, with functions of enhancing immunogenicity of unmodified, unpurified naked RNA, promoting DC maturation and enhancing cytotoxic T lymphocyte response, and has been used in several clinical trials [3]. The fourth is granulocyte–macrophage colony-stimulating factor (GM-CSF), which is considered to enhance the local recruitment and activation of DCs and promote tumor antigen presentation, also activate other cells in the immune response [116]. The fifth category is some mRNA carriers, such as cationic lipids and protamine. The use of cationic lipids DOTAP/DOPE loaded mRNA can act as an adjuvant and induce more pro-inflammatory cytokines and type I IFN secretion than bare mRNA injection alone [117]; protamine-mRNA complex can act as an adjuvant by activating TLR7/8 [118].

## Delivery approaches

In order to efficiently deliver mRNA into cells and maintain high translation efficiency, scientists have made unremitting efforts to explore various delivery methods, including direct injection of naked mRNA, delivery of naked mRNA using physical methods such as electroporation and gene gun, or delivery through other carriers such as biological nanomaterials [119]. The popular delivery methods are discussed below.

## Ex Vivo Loading of DCs Delivery System

DCs is an important delivery tool for early vaccine studies and can be efficiently transfected by electroporation with mRNAs that mostly encode viral antigens, cancer-testis antigens, overexpression antigens, and differentiation antigens [120]. Several clinical trials have discovered that DC-loaded mRNAs in vitro can efficiently encode antigens and elicit antigen-specific T cell responses [121]. The most commonly used DCs in clinical trials is monocyte-derived DCs which derived from peripheral blood mononuclear cells (PBMCs) or leukocyte [121].Given that such DCs is distinct from the steady-state DC subsets present in vivo, it is unclear which primary DCS are the best candidates [121]. Another reason that hinders the application of DC-based delivery is that the preparation process involves cell isolation, purification, transfection, and maturation, which is time-consuming and costly, making it difficult to meet the urgent therapeutic needs of some patients [122].

## Lipid nanoparticles

LNPs are one of the most popular and widely used tools for the mRNA delivery, which are mainly composed of ionizable amino lipids, polyethylene glycol, phospholipids and cholesterol. The related progress ofresearch has been discussed in detail in several recent reviews [123, 124]. Briefly, the basic process is that negatively charged mRNA molecules can be delivered stably wrapped in the inner core by electrostatic interaction with positively charged lipids, free from enzymatic degradation by extracellular RNases and endosomes [125]. Upon reaching the antigen-presenting cells, lipid nanoparticles can enter the cells through various mechanisms such as macrocytic drinking and endocytosis, and subsequently stay in the endosomal compartment. In the endosomes, positively charged lipids may interact and fuse with the negatively charged endosomal membrane, resulting in the rupture of the endosomal membrane, allowing mRNA molecules to leak from LNPs and endosomes into the cytoplasm to perform further translation functions [123, 126]. A number of preclinical and clinical trials have confirmed that LNPs are promising mRNA vaccine carriers that can effectively activate the immune response, and continuously improving technologies have enabled LNPs to exhibit more complex structures and enhanced physical stability, bringing fruitful results for the innovation of vaccine delivery systems [124].

## Polymer-based carrier

Polyplexes or polymer-based nucleic acid carriers as another commonly used tool for the mRNA delivery, are similar to LNPs in that polyplexes can also be positively charged and transfect mRNA through electrostatic interactions with mRNA [73]. Early commonly used polymer delivery materials include polyethyleneimine (PEI), poly(l-lysine) (PLL) and poly(amidoamine) (PAMAM). Although PEI was relatively widely used as a delivery system for mRNA vaccines, the cytotoxicity problems associated with its high cationic charge density and degradation problems limited its further application [7]. Subsequent development of multimeric delivery tools has targeted materials with biodegradable structures, the most applied of which is poly-β-amino ester (PBAE), which further improves its serum stability and the transfection efficiency when incorporating lipids [127–129]. Other materials include amino polyesters, which are also biodegradable and are characterized for tissue-selective mRNA delivery [119]. One study compared the recently developed polymer pABOL with LNPS in many aspects, and found that LNPs were superior to pABOL in terms of cellular response to SARS-COV-2 and the activation and reactivity of Th2 [130]. Although it cannot be asserted that LNPs are necessarily more suitable as delivery vehicles than polymers, and a more multidimensional comparison of different classes of delivery vehicles is needed, it is true that polymer-based delivery vehicles need to be more fully developed to enrich their delivery performance.

## Peptide-based delivery

In addition to the previously described delivery vectors, peptides can also be used for mRNA delivery because of the ability of certain positively charged amino acids to adsorb mRNA through electrostatic interactions and the ability of cell-penetrating peptides (CPPs) to rapidly internalize across biological membranes [131, 132]. Changing the ratio of charged amino/phosphate groups in the peptide can regulate particle size and thus alter encapsulation efficiency, while modulating the mass ratio of the peptide, such as protamine to mRNA, can alter the protein expression and cytokine secretion levels [131, 133]. Protamine is a typical peptide carrier that, in addition to stably binding mRNA for delivery, also serves as an adjuvant to activate TLR7/8 to induce an innate immune response [133]. To overcome the previous shortcomings, many researches try to optimize the structure of protamine through the rational design of protamine nanocapsules incorporated with oily nuclei and polyethylene glycol, and the preparation of fusion peptide Xentry by combining protamine with a short CPP et al. [134, 135].

## Virus-like Replicon Particles (VRP)

Viral vectors are another option for the mRNA delivery, among which adenovirus, alphavirus, Sendai virus, flavivirus and picornaviruses have been used for the mRNA delivery [79]. However, most adenovirus vaccines are developed for viral diseases, as well as some bacterial and parasitic diseases and monogenic disorders [136, 137]. At present, the common viral vectors used for tumor antigen mRNA delivery usually appear in the form of VRP to synthetic SAM, which is mostly originated from alphavirus [64]. Compared with traditional mRNA, SAM uses ORFs encoding antigens of interest to replace the ORFs of the viral genome, while retaining the viral genome encoding non-structural replicating specific proteins [136]. The basic principle is that self-replicating replicons are wrapped into VRP by co-transfecting cells in vitro with helper RNA which encodes a viral structural protein, and then encapsulating SAM encoding tumor antigens, so as to achieve the purpose of simulating viral replication without forming infectious particles [138]. This vector has the advantage of increasing the level of RNA replication as well as triggering an innate immune response and promoting the maturation of dendritic cells; however, it also has the disadvantage of inducing neutralizing antibodies against viral surface proteins, thus impeding its application [139, 140]. Recently, a novel RNA delivery method -SEND system has been developed, which uses human self-expressed protein PEG10 combined with RNA to assemble virus-like particles, which has the advantages of safety [141]. The use of self-expressed proteins to bind RNA for delivery can effectively avoid rejection. The developed tools to explore the interaction between mRNA and protein, such as PMTRIPs [142], undoubtedly provide effective help for exploring and developing such proteins as delivery carriers.

## Cationic nanoemulsions (CNEs)

CNEs are another delivery vehicle whose composition, preparation methods, physicochemical properties, and biological behavior have been summarized in a review [143]. Briefly, CNE is a dispersion of an oil phase in an aqueous phase, consisting of an oil core of vegetable or semi-synthetic origin, stabilized by a cationic surfactant [143]. Studies have also been carried out to investigate the stability, toxicity, and biodistribution of CNE, and the results of these studies confirm its stability, but toxicity has not been consistently concluded in different models [144–146].

## Administration routes

The degree of antigen uptake, expression and presentation by antigen presenting cells varies with the route of injection, which will alter the magnitude and quality of neoantigen-specific CD8 + T cells and ultimately affect the strength, speed, and duration of immune responses and side effects [147]. Conventional delivery routes include intradermal, intra tumoral, intranodal, intravenous, subcutaneous and intranasal [96]. Intramuscular injection is one of the commonly used administration routes, which has the characteristics of easy operation, well tolerance, flexible injection dose and less side effects at the injection site [148]. In an experiment with mice, the researchers compared the efficiency of LNPs- mRNA delivery by six different injection routes, and intramuscular injection showed good performance in duration of mRNA translation and protein expression level [149]. In a non-human primate experiment, the researchers used a non-invasive method of a dual radionuclide near-infrared probe to monitor the temporal and spatial trafficking characteristics of the vaccine by intramuscular injection, providing valuable help for accurately evaluating the dose, injection route and biological distribution of the vaccine [150, 151]. Many ongoing clinical trials also choose intramuscular injection to deliver LNPs-mRNA [152]. In view of the advantages and disadvantages of each vaccination route according to the disease own characteristics, monitoring and studying the spatio-temporal dynamics of the vaccine can reasonably select the vaccination route to ensure that the vaccine efficacy can be maximized.

## Advances in clinical trials of mRNA tumor vaccines

To date, the majority of registered clinical trials of mRNA tumor vaccines are under phase I and phase II, which aim to evaluate the safety, tolerability, and efficacy of the vaccines (Table 2 and Table3, Supplementary Table 1). Early clinical trials of mRNA tumor vaccines, mostly targeted tumor-associated antigens**.** Several melanoma studies observed enhanced antigen-specific immune responses and clinical outcomes when the vaccine was combined with other immunotherapies [153–155]. However, in this study (NCT02410733), no advantage was observed for the combination of anti-PD1 therapy, as the number of patients who responded (partial + complete responses) to the TAA vaccine FixVac alone was 10/25, while the number of responses in vaccinated patients additionally treated with anti-PD1 therapy was 8/17 [156]. In addition, when TAA-DC vaccine was combined with chemotherapy agents, no stronger tumor-specific T cell responses or improved clinical outcomes were observed in combination therapy compared with DC vaccine monotherapy, no matter in either melanoma or prostate cancer studies [157, 158]. However, it is difficult to determine from several clinical trials whether the combination therapy can produce an enhanced antitumor effect, as various factors such as different diseases and patient stages will have an impact on this. Equally, it is difficult to compare the actual effectiveness of different delivery methods when the observed indicators are different. Therefore, it is necessary to expand the included subjects and unify intervention and observation indicators so as to objectively evaluate the effect of combined therapy and different delivery materials. With the rise of neoantigen screening methods, personalized vaccines became a hot topic in tumor vaccine development. However, the development of mRNA vaccines tailored to patient's genetic mutation is still in its infancy. The first human neoantigen mRNA vaccine was developed and tested in melanoma patients. The vaccine contained 20 different neoepitopes, 60% of which were immunogenic, and 75% of the patients had a progression-free survival of 27 months [159]. In a study that mRNA vaccines targeting poly-neoepitopes against gastrointestinal cancer, a total of 15.7% of the potential neoantigen induced specific T cell immunity which mainly consisting of CD4 + T cell response (59%), while no objective clinical responses in the 4 treated patients were observed [160]. Many clinical trials of personalize vaccines have been conducted against multiple tumors simultaneously, of which the mRNA sequence was designed to encode multiple neoepitopes and delivery vehicles was chosen liposomal nanoparticles which are currently of great interest. More and more companies are participating in the mRNA tumor vaccine development, which has greatly expanded the market size of mRNA vaccine [161]. For instance, mRNA-5671, an mRNA cancer vaccine developed by Modena and Merck, which encodes four key KRAS mutants and delivered via LNPs, is currently under evaluation for safety and tolerability in a Phase I clinical trial. In addition to encoding specific antigens or tumor-associated antigens, there are also vaccines which mRNA encoding immunomodulatory factors. One such vaccine, mRNA-2752, is an mRNA vaccine encoding three immunomodulatory factors and is currently being evaluated in Phase I and II clinical trials for safety and tolerability alone and in combination with a fixed dose of durvalumab in patients with relapsed/refractory solid tumors or lymphomas.

**Table 2:** Clinical trials of mRNA vaccine against single tumor

Registration number	Status	Diseases	Phase	Encoding mRNA	Adjuvants	Delivery Tools	Additional therapies	Outcomes	ref
Registration number	Status	Diseases	Phase	Encoding mRNA	Adjuvants	Delivery Tools	Additional therapies	Outcomes	ref
NCT03394937	Terminated	Melanoma	I	TAA: gp100, tyrosinase, MAGE-A3, MAGE-C2, PRAME	TriMix	Synthetic naked mRNA	-	Immunologically active cells against cancer were activated and expected potential risks are non-serious and related to the local administration of the product	[53]
NCT01066390	Completed	Melanoma	IB	TAA: either gp100, MAGE-A3, MAGE-C2 or tyrosinase	TriMix	DCs-based	-	15 patients tolerated administration of the vaccine well. Four durable objective tumor responses were observed during a follow-up over 2 years. Antigen-specific skin infiltrating lymphocytes were detected in 6/12 patients	[154]
NCT01676779	Completed	Melanoma	II	No exact details	TriMix	DCs-based	-	71% of patients in TriMix cohort were alive and free of disease compared to 35% in the control cohort after 1 year into the group. 9/21 patients in TriMix cohort experienced a non-salvageable melanoma recurrence compared to 14/20 in the control cohort after a median follow-up of 53 months	[181]
NCT01302496	Completed	Melanoma Stage III/ IV	II	TAA: tyrosinase, gp100, MAGE-A3, or MAGE-C2	TriMix	DCs-based	Ipilimumab	T-cell responses against antigens were detected in 12/15 patients. Treated patients resulted in an OS of 28% and a PFS of 18% during follow-up over 5 years	[155]
NCT02410733	Active, not recruiting	advanced melanoma	I	TAA: NY-ESO-1, Tyrosinase, MAGE-A3, TPTE	-	Cationic liposomes	anti-PD1 therapy	Exploratory interim analysis showed that long-lasting antigen-specific CD4/8 + T cell responses were induced and regression of lesions was observed in multiple patients within 300 days of initiation of treatment	[156]
NCT02035956	Completed	Melanoma Stage III/ IV	I	TSA: poly-neoepitope; RBL001/RBL002	-	Naked mRNA	-	T cell response were detected in 60% selected neoepitopes. 75% of the patients had a progression-free survival of 27 months	[159]
NCT01684241	Terminated	Metastatic melanoma	I	TAA: MART, tyrosinase, gp100, MAGE-3	-	DCs-based	GITR-L, Anti-CTLA4 mAb	Antigen-specific immune responses against TAAs and survival were enhanced when vaccines combing with GITR-L and anti-CTLA-4 mAb	[153]
NCT00978913	Completed	Melanoma	I	TAA: hTERT, survivin, p53	-	DCs-based	Cyclophosphamide	Immune responses were detected in 6/17 patients by IFNγ ELISpot and 4/17 patients by proliferation assay. 9/22 treated patients achieved disease stabilization, 3/18 evaluable patients experienced tumor shrinkage	[182]
NCT00243529	Completed	Melanoma Stage III/ IV	I/II	TAA: Tyrosinase, gp100	-	DCs-based	-	TAA-specific CD4/CD8 + T cell responses were detected in 17/26 stage III patients and 11/19 stage IV patients. Stage IV patients with TAA-specific CD8 + T cell responses had a median overall survival of about 12 months longer than those who without response	[183]
NCT02285413	Completed	Melanoma Stage III/ IV	II	TAA: Tyrosinase, gp100	-	DCs-based	Cisplatinum	44% antigen-specific CD8 + T cells and 28% functional T cell responses were detected in skin infiltrating lymphocytes from vaccinated patients with cisplatin. The relevant data were 67% and 19% for those vaccinated alone	[157]
NCT00204607	Completed	Melanoma Stage III/ IV	I/II	TAA: gp100, Melan-A, MageA3, survivin, MageA1, tyrosinase	GM-CSF	Protamine	-	Vaccine-induced T cells were increased in 2/4 immunologically evaluable patients and a complete response was observed in 1/7 patients with measurable disease	[184]
NCT00678119	Completed	Renal cell carcinoma	II	TAA and immune modulators	CD40L	DCs-based	sunitinib	62% patients experienced clinical benefit (9 partial responses, 4 with stable disease), and five patients (24%) survived for more than 5 years	[185]
NCT01890213	Completed	Colorectal Cancer	I	TAA: CEA	-	VRP	-	CEA-specific humoral immunity was observed in all involved patients. For patients with stage IV cancer, 5-year survival was 17%, (95% CI 6% to 33%). For patients with stage III cancer (n = 12), the 5-year RFS was 75%, (95%CI 40% to 91%)	[186]
NCT00228189	Completed	Colorectal Cancer	I/II	TAA: CEA	-	DCs-based	-	The median PFS after mRNA-loaded DCs treatment was 26 months and median PFS for patients with peptide-loaded DCs treatment was 18 months	[187]
NCT02709616 NCT02808364	Completed	GBM	I	TAA: containing 3–13 different TAAs	-	DC-based	low-dose cyclophosphamide, imiquimod poly I:C, and anti-PD-1 antibody	TAA-specifc CD4 + or CD8 + T cell responses were detected in 2/5 GBM patients. The median survival time was 19 months for the GBM patients	[188]
NCT00846456	Completed	GBM	I/II	GSCs mRNA	-	DC-based	-	For vaccinated patients, the PFS was 694 days; for control group, the PFS was 236 days	[189]
NCT01686334	Recruiting	AML	II	TAA: WT1	-	DC-based	low-intensity chemotherapy	5/10 patients with high WT-1expression showed molecular remission after vaccination, of 2 patients achieved complete remission	[190]
NCT01278940	Completed	Melanoma	I/II	No exact details	-	DCs-based	IL-2	Higher T cell proliferation to transferred DCs (tDC) than mockDC controls were observed in 10/19 patients. tDC-specific response was still detected over 10 weeks after last vaccination in 4/6 patients	[191]
NCT03164772	Completed	NSCLC	I/II	TAA: MAGE-C1、NY-ESO-1、MAGE-C2、survivin、5T4, MUC-1	-	Protamine	Durvalumab, Tremelimumab	Vaccine was well-tolerated, and immune responses against TAAs were detected in majority of patients	[53]
NCT00923312	Completed	NSCLC	I/II	TAA: NY-ESO-1, 5T4, MAGE-C1, MAGE-C2, survivin, MUC-1	-	LNP	-	CV9201 was well-tolerated. Median PFS and OS were 5.0 months (95% CI 1.8–6.3) and 10.8 months (8.1–16.7) since first administration. 2- and 3-year survival rates were 26.7% and 20.7%, respectively	[192]
NCT01915524	Terminated	NSCLC	I	TAA: NY-ESO-1, MAGE-C1, MAGE-C2, survivin, 5T4, MUC-1	-	Protamine	Pemetrexed	80% patients had elevated levels of antigen-specific antibodies and 40% had elevated levels of functional T cells. 46.2% patients achieved stable disease	[193]
NCT01446731	Completed	Prostate cancer	II	TAA: PSA, PAP, survivin, hTERT	-	DCs-based	Docetaxel	PFS and OS were 5.5 and 21.9 months, respectively, for patients receiving docetaxel monotherapy and 5.7 and 25.1 months, respectively, for patients receiving combination therapy	[158]
EudraCT number:2008–003,967-37	Completed	Prostate cancer	I/II	TAA: PSA, PSCA, PSMA, STEAP1	-	protamine	-	A cellular immune response was observed in 25/33 of the patients. median OS was 31.4 months [95% CI: 21.2] for 36 treated patients with metastatic prostate cancer	[194]

*NSCLC* non-small cell lung cancer, *GBM* glioblastoma, *GSMs* Glioma stem cells, *AML* acute myeloid leukemia, *TAA* tumor-associated antigen, *TSA* tumor specific antigen, *LNP* Liposome nanoparticles

**Table 3:** Clinical trials of mRNA vaccine against multi-tumor

NCT number	Status	Diseases	Phase	Encoding mRNA	Adjuvants	Delivery tools	Additional therapies
NCT00529984	Completed	Colorectal Cancer; Breast Cancer; Lung Cancer; Pancreatic Cancer; Colon Cancer	I/II	TAA: CEA		VRP	-
NCT03948763	Active, not recruiting	Neoplasms; NSCLC; Pancreatic Neoplasms Colorectal Neoplasms	I	TSA: KRAS mutation (G12C, G12D, G12V, G13C)	-	LNP	pembrolizumab
NCT03289962	Active, not recruiting	Melanoma; NSCLC; Bladder Cancer; Colorectal Cancer; Tripple negative breast cancer; Renal Cancer; Head and Neck Cancer; Other Solid Cancers	I	TSA: poly-neoepitope	-	Lipoplex	Atezolizumab
NCT03323398	Terminated	Relapsed/Refractory Solid Tumor Malignancies or Lymphoma; Ovarian Cancer	I	OX40L, IL-23, IL-36γ	-	LNP	Durvalumab
NCT03639714	Active, not recruiting	NSCLC; Colorectal Cancer; Gastroesophageal Adenocarcinoma; Urothelial Carcinoma	I/II	TSA: poly-neoepitope			Nivolumab; ipilimumab
NCT00004604	Completed	Breast Cancer; Colorectal Cancer; Extrahepatic Bile Duct Cancer; Gallbladder Cancer; Gastric Cancer Head and Neck Cancer; Liver Cancer; Lung Cancer; Metastatic Cancer; Ovarian Cancer; Pancreatic Cancer; Testicular Germ Cell Tumor	I	TAA: CEA	-	DCs-based	-
NCT03468244	Unknown	Advanced Esophageal Squamous Carcinoma; Gastric Adenocarcinoma; Pancreatic Adenocarcinoma; Colorectal Adenocarcinoma	-	TSA: poly-neoepitope	-	Lipo-polyplex	-

*TAA* tumor-associated antigen, *TSA* tumor specific antigen, *LNP* Liposome nanoparticles, *VRP* virus-like replicon particles, *CEA* carcinoembryonic antigen

## Conclusions

Genetic instability contributes to the development of cancer and shapes its heterogeneity, resulting in differential sensitivity to treatment for tumor patients. With deeper understanding of individual mutant antigens, breakthroughs in sequencing technology and the rise of big data and machine learning, rapid and accurate identification of patient-specific mutations in an economical and inexpensive manner has been achieved. Meanwhile, in vaccine preparation, the application of strategies such as optimized mRNA sequences and improved preparation conditions have broken through the previous bottleneck of instability and strong immunostimulatory property of mRNAs, while the control of vaccine delivery has been enhanced by optimized biomaterials and improved engineering techniques. These technological advances have laid a solid foundation for the widespread implementation of precision medicine represented by personalized mRNA vaccines. However, opportunities always come with challenges. Despite the abundance of tumor mutations, very few mutant peptides have been found to actually trigger anti-tumor responses, and it is still time-consuming and labor-intensive to accurately select neoepitopes from the huge number of predicted epitopes that can actually activate immune responses. On the one hand, studies on the immunogenicity of neoepitopes are limited, and no general rule of immunogenic neoepitopes has been found. Therefore, there is still a big obstacle to identify neoepitopes efficiently through streamlined algorithms and experiments. With regard to vaccine preparation, ex vivo loading of DCs delivery system played an important role in early tumor vaccine delivery. In recent years, biomaterials represented by lipid nanoparticles have been continuously improved and innovated, showing superior performance which makes them favored in the preparation of a new round of mRNA vaccines. But there is a lack of researches directly comparing which delivery vehicle is the most advantageous in terms of safety and delivery efficiency. At present, clinical trials of mRNA tumor vaccines are being actively carried out and are generally in the early stage of development. Clinical trials with reported results, especially personalized mRNA tumor vaccine trials are still few, and treatment efficacy has shown great variation in different patients. So more and in-depth research is needed to continue to explore the most appropriate treatment populations, clinical settings, delivery approach, doses and routes of administration, as well as combination therapy before achieving another breakthrough in tumor immunotherapy and benefiting the masses of tumor patients.

## Supplementary Information

**Additional file 1.**

## Data Availability

Not applicable.

## Abbreviations

TAAs: Tumor-associated antigens
CTAs: Cancer testis antigens
MHC: Major histocompatibility complex
PSA: Prostate specific antigen
TSAs: Tumor specific antigens
MS: Mass spectrometry
FFPE: Formalin fixed paraffin embedded
TAP: Transporter associated with antigen processing
pMHC: Peptide MHC
PDOs: Patient-derived organoids
IST: In situ MHC tetramer
IVT: In vitro transcription
ORF: Open reading frame
UTR: Untranslated region
m7G: 7-Methylgaunosine
ARCA: Anti-reverse cap analog
HBA: Hemoglobin subunit α
HBB: Hemoglobin subunit β
PAMPs: Pathogen-associated molecular patterns
dsRNA: Double-stranded RNA
HPLC: High performance liquid chromatography
ψ: Pseudouridine
m6A: N6-methyladenosine
m5C: 5-Methylcytidine
s2U: 2-Thiouridine
m1ψ: N1-methyl pseudouridine
LNPs: Lipid nanoparticles
CD40L: CD40 ligand
GM-CSF: Granulocyte–macrophage colony-stimulating factor
PEI: Polyethyleneimine
PLL: Poly(l-lysine)
PAMAM: Poly(amidoamine)
PBAE: Poly-β-amino ester
CPPs: Cell-penetrating peptides
VRP: Virus-like replicon particles
SAM: Self-amplified mRNAs
CNEs: Cationic nanoemulsions
LC–MS: Liquid chromatography and mass spectrometry
TCR: T cell receptor
LOH: Loss of heterozygosity
TLR: Toll-like receptor
DCs: Dendritic cells
SNV: Single nucleotide variation
CNV: Copy number variation
MNV: Multi-nucleotide variation
NSCLC: Non-small cell lung cancer
GBM: Glioblastoma
GSMs: Glioma stem cells
AML: Acute myeloid leukemia
