# Supplementary material for: Advances of mRNA vaccine in tumor: a maze of opportunities and challenges
Source: Biomark Res. 2023 Jan 18;11:6. doi: 10.1186/s40364-023-00449-w (PMC9845107; doi:10.1186/s40364-023-00449-w)
Supplement: Supplementary file 1 — Additional file 1. [file 40364_2023_449_MOESM1_ESM.docx]

**Supplementary Table 1. Clinical trials of mRNA tumor vaccines against single tumor without outcomes**

| Registration number | Status | Diseases | Phase | Encoding mRNA | Adjuvants | Delivery Tools | Additional therapies |
| --- | --- | --- | --- | --- | --- | --- | --- |
| NCT00204516 | Completed | Melanoma  Stage III/ IV | I/II | TAA:Melan-A, MAGE-A1, MAGE-A3,gp100, Survivin, and tyrosinase | GM-CSF | Naked mRNA | - |
| NCT03815058 | Active, not recruiting | Melanoma  Stage III/ IV | II | TSA:  poly-neoepitope | - | LNP | Pembrolizumab |
| NCT03897881 | Active, not recruiting | Melanoma | II | TSA:  poly-neoepitope | - | LNP | Pembrolizumab |
| NCT01456104 | Active, not recruiting | Melanoma | I | TAA:  TRP2 |  | DCs-based | - |
| NCT00961844 | Terminated (Logistical problems) | Melanoma | I/II | TAA:  hTERT, survivin | - | DCs-based | Temozolomide |
| NCT00929019 | Terminated (slow accrual) | Melanoma | I/II | TAA:  Tyrosinase, gp100 | - | DCs-based | - |
| NCT01530698 | Completed | Melanoma  Stage III/ IV | I/II | TAA:  Tyrosinase, gp100 | TLR4, CD70 | DCs-based | - |
| NCT03480152 | Terminated | Melanoma | I/II | TSA:  poly-neoepitope |  |  |  |
| NCT00940004 | Completed | Melanoma  Stage III/ IV | I/II | TAA:  Tyrosinase, gp100 | - | DCs-based | - |
| NCT03739931 | Recruiting | NSCLC | II | Immune modulator：OX40L, IL-23, IL-36γ | - | LNP | Durvalumab |
| NCT03948763 | Active, not recruiting | NSCLC | I | TSA: KRAS-G12D, G12V, G13D, G12C | - | LNP | Pembrolizumab |
| NCT04267237 | Withdrawn | NSCLC | II | TSA:  poly-neoepitope | - | LNP | Atezolizumab |
| NCT04998474 | Not yet recruiting | NSCLC | II | TSA:  poly-neoepitope | - | - | Pembrolizumab |
| NCT01153113 | Withdrawn | Prostate cancer | I/II | TAA: hTERT | - | DCs-based | - |
| NCT01197625 | Active, not recruiting | Prostate cancer | I/II | TAA: hTERT, Survivin | - | DCs-based | - |
| NCT04382898 | Recruiting | Prostate cancer | I/II | TAA: no exact details | - | liposomes | Cemiplimab |

**Supplementary Table 1. (continued)**

| Registration number | Status | Diseases | Phase | Encoding mRNA | Adjuvants | Delivery Tools | Additional therapies |
| --- | --- | --- | --- | --- | --- | --- | --- |
| NCT00003432 | Terminated | Breast Cancer | I/II | TAA: CEA | QS21 | DCs-based | - |
| NCT03788083 | Recruiting | Early-stage Breast Cancer | I | Immune modulator：CD70, CD40L, constitutively  active TLR4 | Trimix | Naked mRNA | - |
| NCT03739931 | Recruiting | TNBC | I | Immune modulator：OX40L, IL-23, IL-36γ | - | LNP | Durvalumab |
| NCT02316457 | Active, not recruiting | TNBC | I | TSA and TAA | - | liposomes | - |
| NCT00978913 | Completed | Breast Cancer | I | TAA:  hTERT, survivin, p53 | - | DCs-based | Cyclophosphamide |
| NCT01582672 | Terminated | Renal cell carcinoma | III | TAA and immune modulators | CD40L | DCs-based | sunitinib |
| NCT00003433 | Completed | Colorectal Cancer | I/II | TAA: CEA | - | DCs-based | - |
| NCT04161755 | Active, not recruiting | Pancreatic Cancer | I | TSA:  poly-neoepitope | - |  | Atezolizumab |
| NCT02261714 | Completed | Pancreatic Cancer | I/II |  |  |  |  |
| NCT04573140 | Recruiting | GBM | I | total tumor mRNA and pp65 full length (fl) LAMP | - | DOTAP liposome | - |
| NCT02649582 | Recruiting | GBM | I/II | TAA: WT1 | - | DCs-based | temozolomide |
| NCT02465268 | Recruiting | GBM | II | TAA: pp65-shLAMP | GM-CSF | DCs-based | - |
| NCT03688178 | Recruiting | GBM | II | TAA: pp65-shLAMP | - | DCs-based | temozolomide |
| NCT02529072 | Completed | GBM | I/II | TAA:  pp65-shLAMP | - | DCs-based | nivolumab |
| NCT03615404 | Completed | GBM | I | TAA:  pp65-flLAMP | GM-CSF | DCs-based | - |
| NCT00626483 | Completed | GBM | I | TAA:  CMV pp65-LAMP | GM-CSF | DCs-based | basiliximab |
| NCT00890032 | Completed | GBM | I | BTSCs mRNA | - | DCs-based | - |

**Supplementary Table 1. (continued)**

| Registration number | Status | Diseases | Phase | Encoding mRNA | Adjuvants | Delivery Tools | Additional therapies |
| --- | --- | --- | --- | --- | --- | --- | --- |
| NCT01334047 | Terminated | Ovarian Cancer | I /II | TAA:  hTERT, survivin | - | DCs-based | - |
| NCT04163094 | Active, not recruiting | Ovarian Cancer | I | TAA | - | liposome | neo-adjuvant chemotherapy |
| NCT00834002 | Completed | AML | I | TAA: WT1 | - | DCs-based | - |
| NCT00510133 | Completed | AML | II | TAA:  hTERT, LAMP | - | DCs-based | - |
| NCT00514189 | Terminated | AML | I | AML lysate plus mRNA (No exact details) | - | DCs-based | - |
| NCT03083054 | Active, not recruiting | AML | I /II | TAA: WT1 | - | DCs-based | - |
| EudraCT: 2010-018770-20 | Ongoing | Prostate cancer | I/II | TAA: hTERT, surviving | - | DCs-based | - |

TAA: tumor-associated antigen; TSA: tumor specific antigen; LNP: liposome nanoparticles. VRP: virus-like replicon particles; NSCLC: non-small cell lung cancer; TNBC: triple-negative breast cancer; AML: acute myeloid leukemia
